# Supplementary material for: Problematic Internet Use: Measurement and Structural Invariance Across Sex and Academic Year Cohorts
Source: Eur J Investig Health Psychol Educ. 2025 Jul 22;15(8):145. doi: 10.3390/ejihpe15080145 (PMC12385649; doi:10.3390/ejihpe15080145)
Supplement: Supplementary file 1 [file ejihpe-15-00145-s001.zip › ejihpe-3726144-supplementary.pdf]

Problematic Internet Use: Measurement and Structural Invariance Across Sex and Academic Year Cohorts

Mateo Pérez-Wiesner, Kora-Mareen Bühler, and Jose Antonio López-Moreno

Supplementary Material

This supplementary document includes additional figures corresponding to the structural equation models (SEMs) applied to each domain of digital media use—internet, messaging, social networking, and video games—presented by academic year and by sex. The figures illustrate the relationships between the independent variables—negative and positive emotion regulation (NER and PER), compulsive use (CU), and cognitive preoccupation (CC)—and their respective negative consequences, modeled as latent constructs.

All models were estimated using robust maximum likelihood (MLR) and display standardized regression weights (β) for the structural paths. The most relevant coefficients are those linking the independent latent variables (NER, PER, CU, CC) with each other and with the dependent latent variable (negative consequences). These paths reflect the hypothesized predictive associations central to the study. In contrast, the arrows connecting each latent variable to its observed indicators represent measurement errors and are not informative for interpreting the conceptual relationships.

These graphical representations support the statistical results reported in the main article, confirming the consistency of the structural patterns across sex and academic year groups.

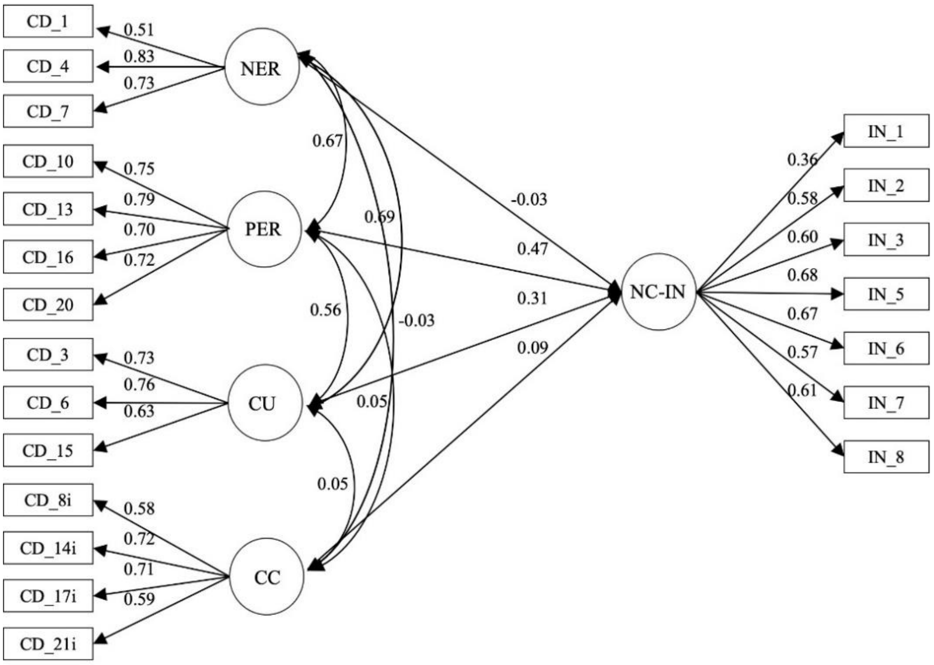

Figure S1. Structural model for negative consequences of internet use by academic year.

Relationships between NER, PER, CU, and CC, and the negative consequences of internet use (NC-IN). Standardized regression coefficients are shown. Separate models are presented for each academic year (1st to 4th grade of secondary education).

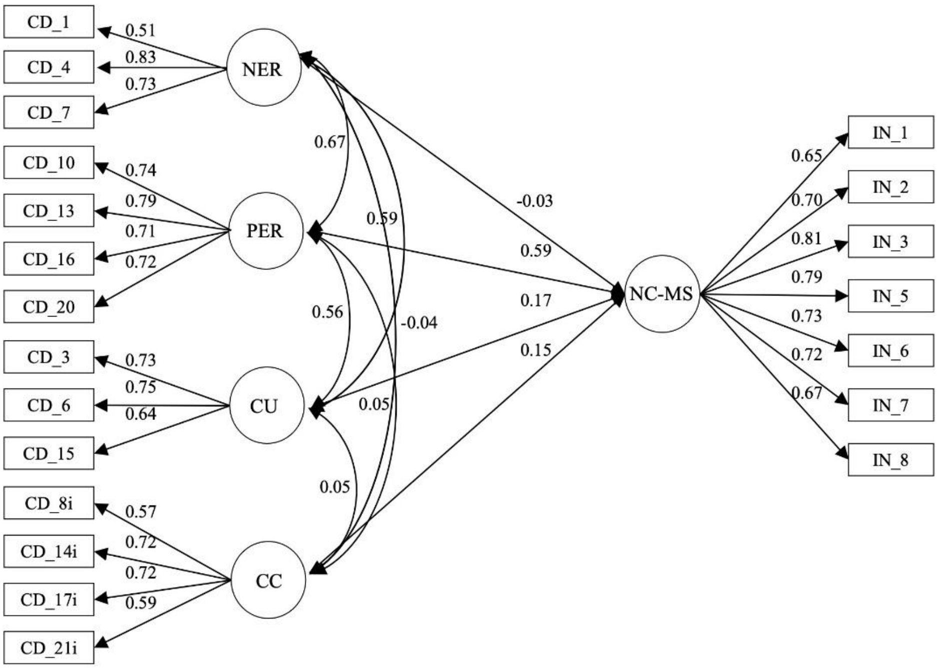

Figure S2. Structural model for negative consequences of instant messaging use by academic year.

Depicts associations between the four predictors (NER, PER, CU, CC) and problematic messaging use (NC-MS). Similar patterns are observed across educational levels, with a progressive increase in some effects at higher grades.

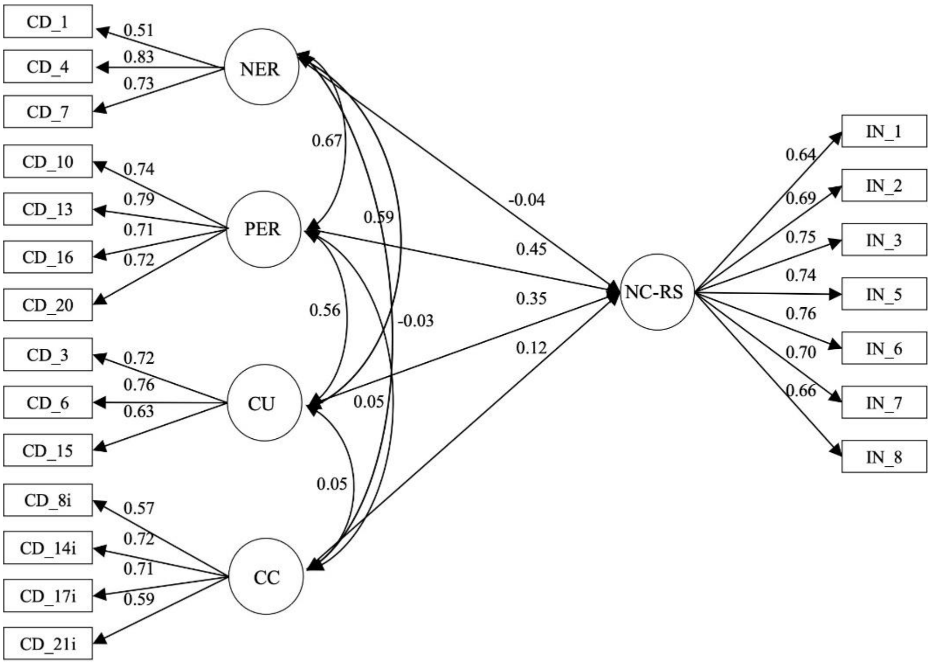

Figure S3. Structural model for negative consequences of social networking use by academic year.

Illustrates the prediction of NC-RS by NER, PER, CU, and CC. Minor variations in regression weights are observed across academic years, while the general relational structure remains stable.

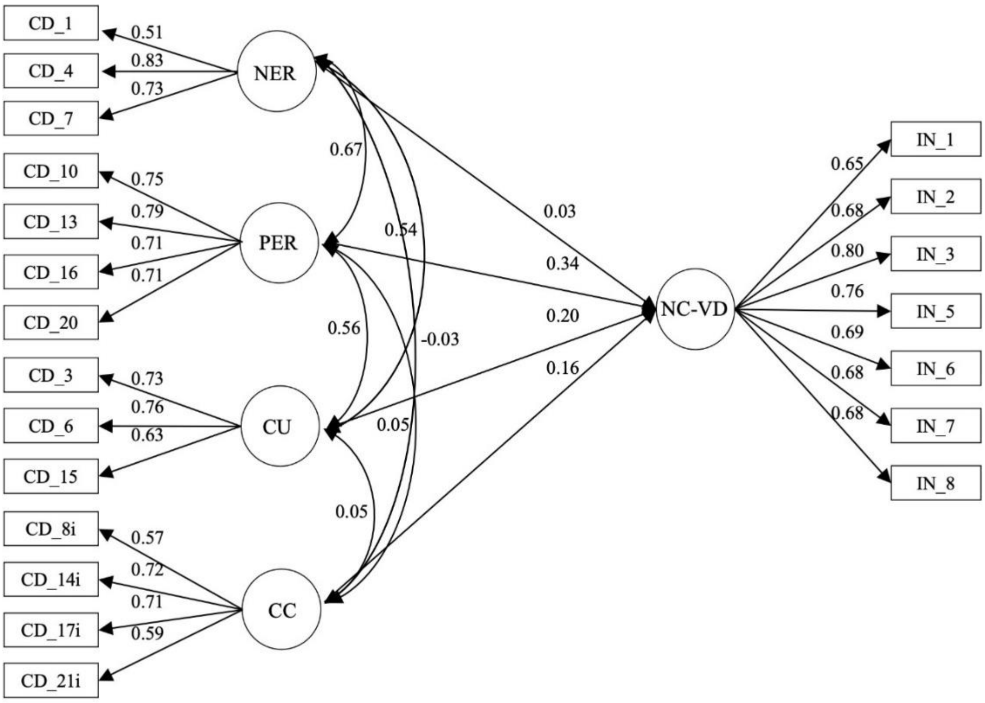

Figure S4. Structural model for negative consequences of video game use by academic year.

Shows how NER, PER, CU, and CC explain negative consequences related to video games (NC-VD). Higher contributions of CU and CC are observed in upper academic levels, especially in 3rd and 4th grades

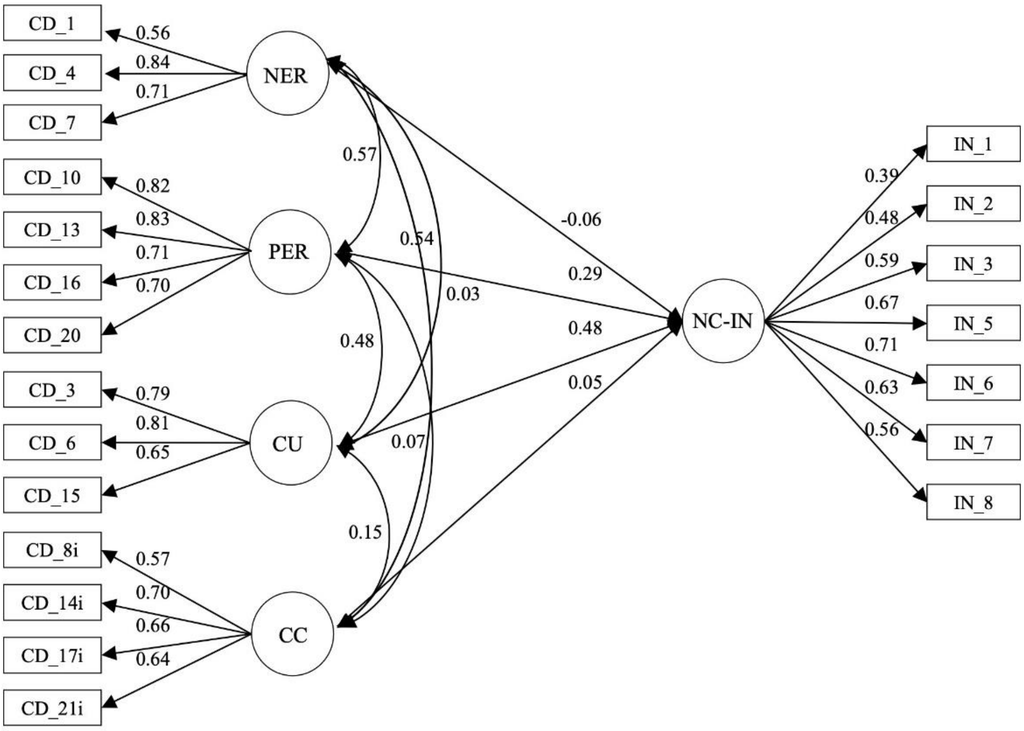

Figure S5. Structural model for negative consequences of internet use by sex.

Displays the relationships between NER, PER, CU, and CC, and negative consequences of internet use (NC-IN) in male and female adolescents. Standardized regression weights are presented separately for each sex

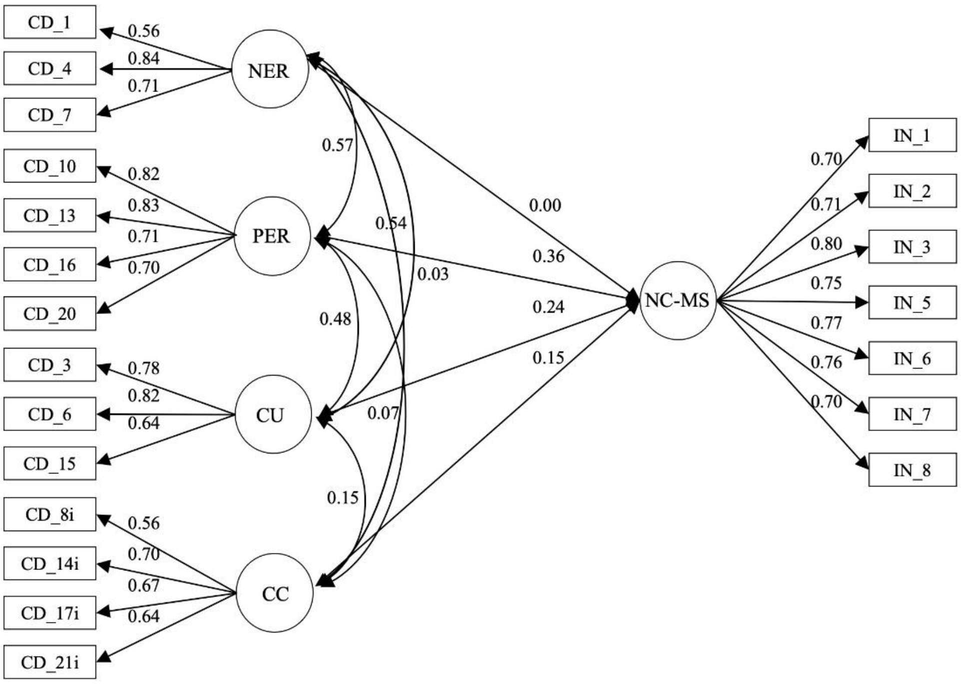

Figure S6. Structural model for negative consequences of instant messaging use by sex.

Shows the SEM results for messaging use (NC-MS) across sexes. Similar paths and strengths of associations are observed between groups, supporting the measurement and structural invariance findings.

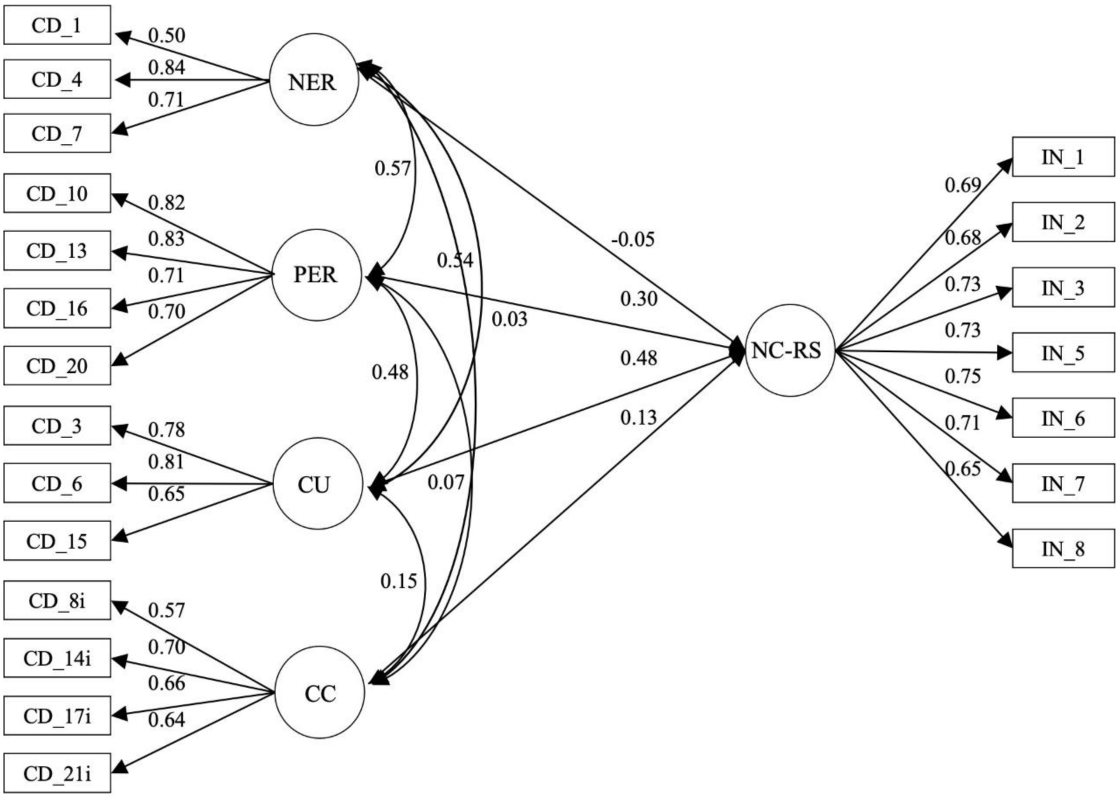

Figure S7. Structural model for negative consequences of social networking use by sex.

SEM models for NC-RS are depicted separately for male and female participants. The figure illustrates comparable patterns of influence across predictors.

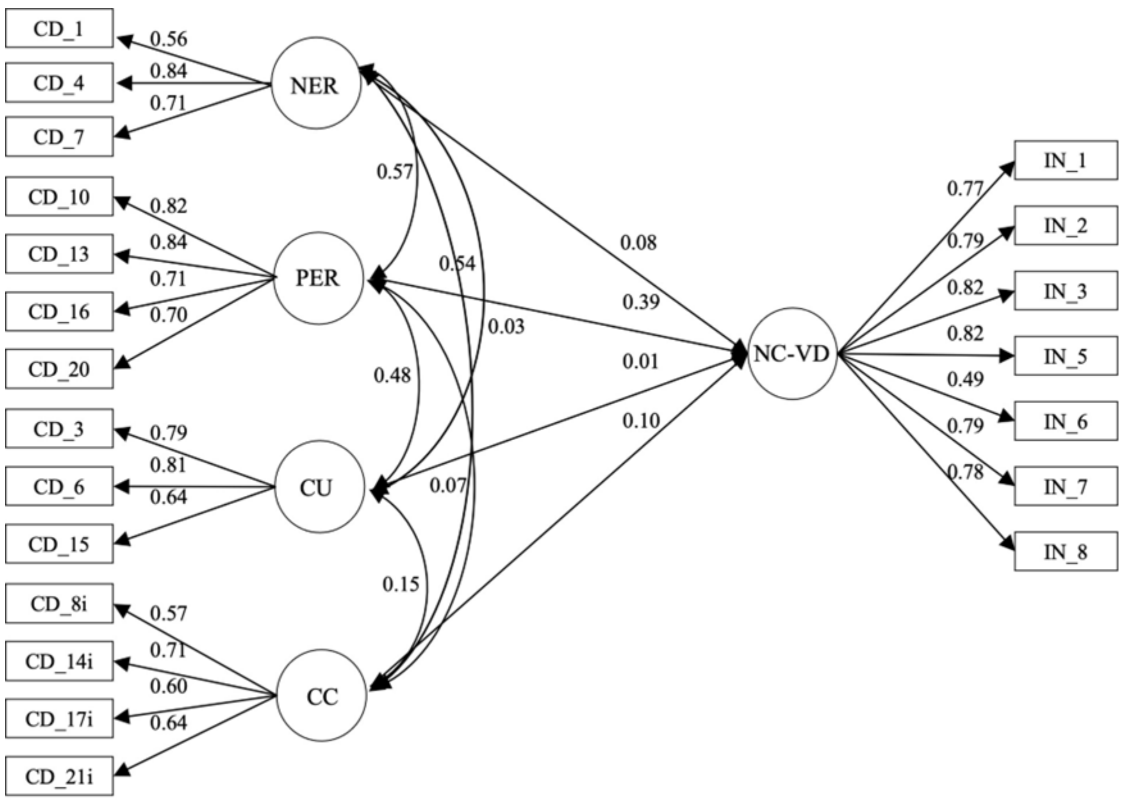

Figure S8. Structural model for negative consequences of video game use by sex.

Illustrates SEM relationships between ER variables and NC-VD in boys and girls. Higher path coefficients for compulsive use and cognitive preoccupation are observed in male participants, although the structural pattern remains stable.
